# Supplementary material for: Formation of synthetic RNA protein granules using engineered phage-coat-protein -RNA complexes
Source: Nat Commun. 2022 Nov 10;13:6811. doi: 10.1038/s41467-022-34644-4 (PMC9649756; doi:10.1038/s41467-022-34644-4)
Supplement: Supplementary file 3 — Description of additional Supplementary File [file 41467_2022_34644_MOESM3_ESM.pdf]

Description of additional Supplementary files

Supplementary Data 1: Sequences of the slncRNA molecules used in the experiments
