# Supplementary material for: Formation of synthetic RNA protein granules using engineered phage-coat-protein -RNA complexes
Source: Nat Commun. 2022 Nov 10;13:6811. doi: 10.1038/s41467-022-34644-4 (PMC9649756; doi:10.1038/s41467-022-34644-4)
Supplement: Supplementary file 5 — Reporting Summary [file 41467_2022_34644_MOESM5_ESM.pdf]

nature portfolio

Corresponding author(s): Roee Amit

Last updated by author(s): Oct 19, 2022

Reporting Summary

Nature Portfolio wishes to improve the reproducibility of the work that we publish. This form provides structure for consistency and transparency in reporting. For further information on Nature Portfolio policies, see our Editorial Policies and the Editorial Policy Checklist.

Statistics

For all statistical analyses, confirm that the following items are present in the figure legend, table legend, main text, or Methods section.

n/a	Confirmed	
☐	☒	The exact sample size (n) for each experimental group/condition, given as a discrete number and unit of measurement
☐	☒	A statement on whether measurements were taken from distinct samples or whether the same sample was measured repeatedly
☐	☒	The statistical test(s) used AND whether they are one- or two-sided<br>*Only common tests should be described solely by name; describe more complex techniques in the Methods section.*
☐	☒	A description of all covariates tested
☐	☒	A description of any assumptions or corrections, such as tests of normality and adjustment for multiple comparisons
☐	☒	A full description of the statistical parameters including central tendency (e.g. means) or other basic estimates (e.g. regression coefficient) AND variation (e.g. standard deviation) or associated estimates of uncertainty (e.g. confidence intervals)
☐	☒	For null hypothesis testing, the test statistic (e.g. F, t, r) with confidence intervals, effect sizes, degrees of freedom and P value noted<br>*Give P values as exact values whenever suitable.*
☒	☐	For Bayesian analysis, information on the choice of priors and Markov chain Monte Carlo settings
☒	☐	For hierarchical and complex designs, identification of the appropriate level for tests and full reporting of outcomes
☐	☒	Estimates of effect sizes (e.g. Cohen's d, Pearson's r), indicating how they were calculated

Our web collection on statistics for biologists contains articles on many of the points above.

Software and code

Policy information about availability of computer code

Data collection

Microscopy control and data acquisition was performed using Nikon NIS-Elements version 4.20.02 (build 988) 64 bit
Super resolution microscope control and data acquisition was performed using ZEN Black version 3.3.89

Data analysis

Custom data analysis scripts were developed and tested on Matlab 2021a 64-bit with the following additions:
- Curve Fitting Toolbox
- Image Processing Toolbox
- Statistics and Machine Learning Toolbox

In addition, ImageJ FIJI (version 1.53c) was used to read microscopy image files and convert to the formats required for the custom built Matlab scripts. The Mosaic suite plugin was used to detect bright spots,
All scripts were built and tested in a Microsoft Windows 10 version 21H1 environment.

Provided custom Matlab scripts developed as part of this work:
- read_tables.m
- collect_intensity_data.m
- generate_movArray.m
- collect_statistical_data.m
- get_position.m
- runLengthEncode.m

- plot_tracks_custom.m
- plot_2_channel_measurements.m

- plot_tracks_custom_subplot_version.m
- fit_data_to_poisson.m
- poisson_sub_func.m
- violin.m
- Generate_plots_for_paper.m

- fca_readfcs.m
- GetFiles.m
- GetMap.m
- natsort.m
- natsortfiles.m
- ReadPlate.m

Code availability: All original code used in this paper, including tutorials and sample data is available from: https://github.com/naorgk/slncRNA_Analysis.

For manuscripts utilizing custom algorithms or software that are central to the research but not yet described in published literature, software must be made available to editors and reviewers. We strongly encourage code deposition in a community repository (e.g. GitHub). See the Nature Portfolio guidelines for submitting code & software for further information.

Data

Policy information about availability of data

All manuscripts must include a data availability statement. This statement should provide the following information, where applicable:

- Accession codes, unique identifiers, or web links for publicly available datasets
- A description of any restrictions on data availability
- For clinical datasets or third party data, please ensure that the statement adheres to our policy

All datasets used in this paper are available from: https://github.com/naorgk/slncRNA_Analysis. (DOI: https://zenodo.org/badge/latestdoi/539529640) All bacterial plasmids constructed for this work are available upon request. Source data are provided with this paper.

Human research participants

Policy information about studies involving human research participants and Sex and Gender in Research.

Reporting on sex and gender	N/A
Population characteristics	N/A
Recruitment	N/A
Ethics oversight	N/A

Note that full information on the approval of the study protocol must also be provided in the manuscript.

Field-specific reporting

Please select the one below that is the best fit for your research. If you are not sure, read the appropriate sections before making your selection.

[x] Life sciences [ ] Behavioural & social sciences [ ] Ecological, evolutionary & environmental sciences

For a reference copy of the document with all sections, see nature.com/documents/nr-reporting-summary-flat.pdf

Life sciences study design

All studies must disclose on these points even when the disclosure is negative.

Sample size	No sample size calculation was performed.<br>Sample sizes was deemed sufficient when new data collected would not drastically alter the median and standard deviation of all calculated observables (i.e., amplitudes, durations and median fluorescence values).
Data exclusions	No data was excluded from the analysis

Replication	The experiments described in the paper were taken on separate days, using different batches of in-vitro transcribed RNA and different batches of bacterial cultures. All attempts at replication were successful.
Randomization	Bacterial cultures were allocated into experimental groups based on the identity of the RNA molecules they express. Covariates were controlled by using an identical expression system for each RNA molecule used (same expression vector under the same promoter).<br><br>In-vitro RNA molecules were allocated to experimental groups by the nature of their design. Covariates were controlled by ascertaining that all molecules have a similar GC content.
Blinding	Blinding was not relevant to the study, since the analysis for all samples was identical and automated.

Reporting for specific materials, systems and methods

We require information from authors about some types of materials, experimental systems and methods used in many studies. Here, indicate whether each material, system or method listed is relevant to your study. If you are not sure if a list item applies to your research, read the appropriate section before selecting a response.

Materials & experimental systems

n/a	Involved in the study
☒	☐ Antibodies
☒	☐ Eukaryotic cell lines
☒	☐ Palaeontology and archaeology
☒	☐ Animals and other organisms
☒	☐ Clinical data
☒	☐ Dual use research of concern

Methods

n/a	Involved in the study
☒	☐ ChIP-seq
☐	☒ Flow cytometry
☒	☐ MRI-based neuroimaging

Flow Cytometry

Plots

Confirm that:

☒ The axis labels state the marker and fluorochrome used (e.g. CD4-FITC).

☒ The axis scales are clearly visible. Include numbers along axes only for bottom left plot of group (a 'group' is an analysis of identical markers).

☒ All plots are contour plots with outliers or pseudocolor plots.

☒ A numerical value for number of cells or percentage (with statistics) is provided.

Methodology

Sample preparation	E. coli BL21 cells expressing the relevant constructs were grown over night in LB at 37 degrees 250 RPM with antibiotics (AMP,CM) and induction where needed (IPTG for slncRNA expression; C4-HSL for tdPCP-mCherry expression)<br>Cells were diluted 1:100 and allowed to grow again to an optical density of 0.3. Then, cells were diluted 1:10 in 1xPBS and measured in triplicate using a 96-wells plate.
Instrument	MACSquant VYB, Miltenyi Biotec
Software	Custom built Matlab scripts have been uploaded to the github repository in the link: https://github.com/naorgk/slncRNA_Analysis
Cell population abundance	Live cells values were >99% for samples and controls. Single cells comprised >99% of the populations of both samples and controls. mCherry=positive cells were >99% for the positive samples and <1% for the negative control.
Gating strategy	A FSC-height trigger was set at 10, and cells were sorted through the following gating process:<br>Live cells (live gate): events that fall within the FSC_AxSSC_A gate were considered live cells<br>Single cells (single gate): of events that were live gate-positive, events that fall within the SSC_AxSSC_H gate were considered single cells<br>mCherry-positive cells (mCherry-pos gate): of events that were single gate-positive, mCherry values were histogramed and a threshold for positive mCherry signal was set based on a value in the negative control sample that leaves around 0.1% mCherry-positive cells.

☒ Tick this box to confirm that a figure exemplifying the gating strategy is provided in the Supplementary Information.
